# Determinants of jaundice severity in neonates admitted at a Teaching Hospital in Ghana

**DOI:** 10.1371/journal.pone.0325003

**Published:** 2025-06-05

**Authors:** Naomi Safo-Mensah, Olivia Amfo-Swanzy, Joyce Ashong, Emmanuel Okai, Manuella Faith Amoako, Percy Lomotey, Oheneba Charles Kofi Hagan, George Adjei

**Affiliations:** 1 School of Medical Sciences, University of Cape Coast, Cape Coast, Ghana; 2 Cape Coast Teaching Hospital, Cape Coast, Ghana; 3 Department of Paediatrics and Child Health, University of Cape Coast, Cape Coast, Ghana; 4 Department of Medical Biochemistry, University of Cape Coast, Cape Coast, Ghana; 5 Department of Community Medicine, University of Cape Coast, Cape Coast, Ghana; Shoklo Malaria Research Unit, THAILAND

## Abstract

**Background:**

Severe neonatal jaundice (NJ) is associated with significant morbidity and mortality globally with low and, middle income countries having a greater burden. Increased red blood cell destruction and reduced bilirubin elimination increase the risk of severe NJ development. Severe NJ predisposes the neonates to bilirubin-induced encephalopathy leading to kernicterus-spectrum disorders in the long-term.

**Objective:**

The study was undertaken to determine the risk factors associated with development of significant jaundice in neonates at a teaching hospital in Ghana.

**Materials and methods:**

A retrospective review of patient electronic medical records at the paediatric ward and Neonatal Intensive Care Unit (NICU) at the Cape Coast Teaching Hospital (CCTH) was conducted. Demographics, initial serum bilirubin concentration (total, direct and indirect), full blood count, G6PD status and outcome data were collected. Binary logistic regression models were used to determine the risk factors of NJ severity which was the main outcome. Stata 18.0 software was used for the analysis.

**Results:**

Data on two hundred and ninety-three (293) neonates were extracted of which 247 were used for further analyses after data cleaning. Of these, 30% had significant NJ defined as initial TSB concentration ≥ 213micromol/L. Significant NJ was significantly associated with admission after 24 hours of birth (aOR = 2.45; 95% CI:1.26,4.77; p = 0.009) and full/partial defect G6PD (aOR = 2.21; 95% CI:1.03,4.71; p = 0.041).

**Conclusions:**

Significant NJ is common and significantly associated with admission after 24 hours of life and G6PD full/partial defect at Cape Coast Teaching Hospital.

## Introduction

Neonatal jaundice (NJ) is defined as the visualisation of yellowish colouration of the skin and conjunctiva of the eye resulting from the deposition of unconjugated bilirubin in a newborn [1]. The phenomenon usually presents in newborns with hyperbilirubinaemia due to increased concentration of total serum bilirubin (TSB) [1]. Most newborns will develop NJ with TSB concentration usually above the adult upper limit of normal of 17 micromol/L, however, they are usually physiological. Nonetheless, severe neonatal jaundice (NJ) defined as TSB concentration greater than 342−428 micromol/L could be injurious to the neonate and may result in serious pathologies collectively termed bilirubin-induced neurological disorders (BIND) [2–4]. However, the definition is not applied universally, for example in low- and middle-income countries which carry the greatest burden of the NJ complications and the need for early treatment is paramount, a threshold of greater than 213 micromol/L is mostly applied [2,3]. Increased TSB predominantly results from increased bilirubin production from increased red blood cell turnover from conditions such as glucose-6-phosphate dehydrogenase (G6PD) deficiency, ABO and Rhesus blood group incompatibility, cephalohaematoma, subgaleal haemorrhage, intracranial haemorrhage, polycythaemia and sepsis [5–8]. Additionally, decreased bilirubin clearance as seen in liver immaturity at birth (especially in the preterm) and UDP-glucuronosyltransferase defect syndromes including Crigler-Najjar type I & II, and Gilbert syndromes also result in NJ [6,9].

Neonatal jaundice (NJ) is highly prevalent globally with about 60% of term and 80% of preterm babies diagnosed annually [5,10]. Low- and middle-income countries (LMICs) carry the highest burden of morbidity and mortality mostly because of poor diagnostic and management capacity [11–14]. Increased severity of NJ is associated with poor outcomes with certain factors reported to increase the risk of developing severe NJ [5]. These factors include gestational age < 38 weeks, presence of haemolytic disorders especially G6PD deficiency, haemodynamic instability, bacterial sepsis, hypoalbuminaemia, exclusive breastfeeding, haematoma, uridine diphosphate-glucuronosyltransferase (UGT) genetic polymorphisms [6].

The pathophysiological basis of BIND is the neurotoxic effect of the hydrophobic free unconjugated bilirubin with the ability to traverse the blood-brain barrier and deposits in the brain cells especially the basal ganglia and the brainstem nuclei resulting in apoptosis and necrosis of these cells resulting in encephalopathy [15,16]. In the early stages, the increased TSB concentration is associated with acute bilirubin-induced encephalopathy (ABE) which presents within days to weeks [13]. Clinically, ABE presents in the early stages as lethargy, hypotonia and poor feeding, intermediate stage as hypertonia or hypotonia, irritability, stupor, opisthotonos and high-pitched cry. In the advanced stage, it may present as pronounced retrocollis-opisthotonos posturing, poor feeding and coma [8,13,17–20]. In months to years, the ABE may evolve to chronic bilirubin-induced encephalopathy (CBE) or kernicterus spectrum disorders (KSD). The early phase of CBE, which occurs within the 1^st^ year, is characterised by hypotonia, hyperreflexia and developmental delays. The later phase of CBE presents as a spectrum of disorders including auditory, visual, dental and extrapyramidal disorders [8,13,17,21].

In Ghana, there has been an increasing trend of neonates being diagnosed with NJ from 2015 to 2019 with 10,684 cases reported in 2020 [22,23]. Although the Ghana Health Service reports an NJ rate of 3 in 100 live births, prevalence of 9.7%, 32.9% and 66.7% have been reported from studies at teaching hospitals in the northern and southern parts of Ghana and a municipal hospital in southern part of Ghana respectively [24–26]. Previous reports have identified certain risk factors for developing NJ patients in Ghana including maternal, delivery and neonatal characteristics [25,27]. Knowledge, attitudes and practices (KAP) of NJ among health workers at health facilities are largely adequate, however, among mothers of NJ patients, KAP is currently adequate or inadequate depending on the setting [23,25,28]. Patient and healthcare worker education on risk factors associated with severe NJ is important to reduce morbidity and mortality. Although the long-term neurological effects of NJ have not been thoroughly investigated in the Ghanaian population, NJ was reported to be a major risk factor for the development of cerebral palsy in Ghanaian children [29]. Therefore, the study was undertaken to determine the risk factors associated with the development of severe NJ in neonates who were admitted on account of NJ or developed NJ while on admission at a teaching hospital in Ghana.

## Materials and methods

### Study design

This was a retrospective review of patient medical records at the Paediatric Ward and Neonatal Intensive Care Unit (NICU) of the Cape Coast Teaching Hospital (CCTH).

### Study area

The Cape Coast Teaching Hospital (CCTH) is a 400-bed capacity hospital in the Central Region of Ghana. The hospital provides standard out-patient, in-patient and surgical services, including neonatal intensive care and paediatric services. The Paediatric Ward has 48-bed capacity and there were 1920 admissions in 2023, whereas the NICU has 24 bed capacity and a total of 1049 admissions in 2023.

### Data management

The CCTH database was accessed on 13/04/2024 for secondary data involving neonates between January 2020 and December 2021. The study included neonates who had an initial or a final diagnosis of NJ and were admitted from January 2020 (when the hospital migrated from paper-based to electronic-based records keeping) to December 2021. The following patient information were extracted, neonate: sex, age at admission (hours), presenting complaint, final diagnosis, G6PD status, haematological indices, serum bilirubin concentration at admission (total, conjugated and unconjugated) and outcome (discharged or otherwise). Data were extracted using the Epicollect 5 data extraction instrument (https://five.epicollect.net/) and exported as csv file for data cleaning.

The dependent variable NJ severity was defined as significant when the TSB concentration was ≥ 213 micromole/L and non-signifcant when the values were < 213 micromol/L [2,3]. Gestational age was estimated in weeks, with preterm birth being < 37 weeks and term birth being ≥ 37 weeks gestation (30). Birth weight was categorised as low birth weight (< 2.5 kg), normal birth weight (2.5 – < 4.0 Kg) and high birth weight (macrosomia) (≥ 4.0) [30–32].

### Data analyses

The cleaned secondary data were exported to GraphPad Prism version 10.0.3 (GraphPad Software, USA) and Stata software version 16.0 (StataCorp LLC, USA) for statistical analyses. Categorical variables were reported as frequencies and percentages. Median estimations were used to determine central tendencies and reported with the interquartile ranges. Association analyses were performed for categorical variables using Pearson’s chi-squared test or Fisher’s exact test. Odds ratios were estimated to determine the predictors of NJ severity using bivariable and multivariable logistic regression models and reported as crude (cOR) and adjusted (aOR) odds ratios with 95% CI. All analyses were two-tailed and a *p*-value of less than 0.05 was considered as statistically significant.

Data used for the statistical analyses have been deposited at the Mendeley Data (https://data.mendeley.com) and embargoed for 2 years.

### Ethical concerns

This study was carried out using a secondary dataset from CCTH. Therefore, ethical clearance with informed consent waiver was sought from the CCTH Ethical Review Committee and was granted with the reference number CCTHERC/EC/2023/037. Study participants confidentiality was ensured since unique ID codes were used to identify them.

## Results

### Patient characteristics

Table 1 presents the characteristics of study participants. A total of 293 individual patient data were extracted. Of the 293, 247 of the data were used for further analysis after data cleaning to remove entries with incomplete data. Of the 247 patients, 59.9% (148) were males with 40.1% (99) were females. Approximately 57% (141) of the neonates were admitted within the first 24 hours of birth with the rest, 43% (106) admitted beyond 24 hours after birth. The median age at presentation was 1 day (IQR = 1.0, 3.0) ranging from 1 to 13 days. Most of the neonates, that is 141 (57.1%) were term with the rest being born preterm with a median gestational age of 38 (IQR = 34.2, 40) weeks. The median birthweight was 2.7 Kg (IQR = 2.0, 3.2) with the highest proportion of the patients, that is 55.9% (138) having normal birth weight, followed by 40.1% (99) who were low birthweight and 4.0% (10) who were macrosomic. Two hundred and forty-five (245) of the patients were delivered at a health facility with the remaining two (2) being delivered at home.

**Table 1:** Characteristics of neonate admitted and treated for NJ at Paediatric Ward and NICU at Cape Coast Teaching Hospital during the study period.

Variable	Median (IQR)
Age at admission (days)	1.0 (1.0, 3.0)
Birth weight (kg)	2.7 (2.0, 3.2)
Gestational age (weeks)	38.0 (34.2, 40.0)
Number of days on admission (days)	5.0 (3.0, 8.0)
Initial total bilirubin (micromol/L)	168.2 (120.6, 227.2)
Initial direct bilirubin (micromol/L)	13.4 (10.7, 18.6)
Initial indirect bilirubin (micromol/L)	147.3 (100.3, 200.3)
Haemoglobin concentration (g/dL)	15.6 (13.7, 16.8)
White Blood Cell Count (10^9^/L)	12.4 (9.0, 19.3)
Neutrophil count (10^9^/L) N = 237	6.0 (3.5, 11.0)
Lymphocyte count (10^9^/L) N = 237	4.5 (3.5, 7.6)
Platelet count (10^9^/L) N = 238	218.0 (175.5, 284.6)

The commonest presenting or referral complaints to the clinic were yellowish discolouration of the skin and conjunctiva (147), preterm delivery (97) and respiratory distress (19). Other presenting/referral complaints included suspected sepsis or infection (14) and others (7) including cephalhaematoma, bleeding from umbilical cord, hypoglycaemia, macrosomia and plethora.

The median initial TSB concentration was 168.2 micromol/L (IQR = 120.6, 227.2), unconjugated bilirubin was 147.3 micromol/L (IQR = 100.3, 200.3) and conjugated of 13.4 micromol/L (IQR = 10.7, 18.6). The median haemoglobin was 15.60 g/dL (IQR = 13.70, 16.80). The median white cell count was 12.36 x 10^9^/L (9.02, 19.31). The median (N = 237) for the neutrophil and lymphocyte 12.4 x 10^9^/L (IQR = 9.0, 19.3) and 4.5 x 10^9^/L (IQR = 3.5, 7.6) respectively. The median platelet count was 218.0 x 10^9^/L (IQR = 175.5, 284.6). G6PD status results were available for 201 patients, out of which 79.6% (160) had no defect in the enzyme, 5% (10) had a partial defect and 15.4% (31) had a full defect.

### Association and predictors of NJ severity

Based on the ≥ 213 micromol/L definition for the severity of NJ, 74 (30%) of the neonates had significant NJ, with the rest classified as non-significant. The association between NJ severity and neonatal characteristics is presented in Table 2.

**Table 2:** Association of NJ severity and neonatal characteristics at Paediatric Ward and NICU at Cape Coast Teaching Hospital during the study period.

Variable (N = 247)	Categories	NJ Severity		*p-*value
		Non-significant N = (173)	Significant N (74)	
Age at admission	≤ 24 hours, n (%)	113 (80.1)	28 (19.9)	< 0.001
	> 24 hours, n (%)	60 (56.6)	46 (43.3)	
Sex	Female, n (%)	72 (72.7)	27 (27.3)	0.45
	Male, n (%)	101 (68.2)	47 (31.8)	
Birth weight	Low birth weight, n (%)	76 (76.8)	23 (23.2)	0.15
	Normal birth weight, n (%)	91 (65.9)	47 (34.1)	
	High birth weight	6 (60.0)	4 (40.0)	
Gestational age at birth	Preterm, n (%)	85 (80.2)	21 (19.8)	0.003
	Term, n (%)	88 (62.4)	53 (37.6)	
Place of birth	Home, n (%)	2 (100)	0 (0.0)	1.0
	Health facility, n (%)	173 (70.0)	74 (30.0)	
Management for hyperbilirubinemia	None/ conservative, n (%)	6 (100)	0 (0.0)	0.18
	Phototherapy, n (%)	167 (69.3)	74 (30.7)	
Outcome	Discharged, n (%)	170 (70.2)	72 (29.8)	0.64
	Died, n (%)	3 (60)	2 (40.0)	
G6PD status N = 201	Full/Partial defect, n (%)	21 (51.2)	20 (48.8)	0.009
	No Defect, n (%)	116 (72.5)	44 (27.5)	

There was a statistically significant association between NJ severity and age at admission (p < 0.001) with 19.9% (28 out of 141) of those admitted within the first day of birth having significant NJ and 43.3% (46 out of 106) of those admitted after day 1 having significant NJ. Approximately 20% (21 out of 106) and 38% (53 out of 141) of preterm and term neonates presented with significant NJ with a statistically significant association between the proportions (p = 0.003). As would be expected, 48.8% (20 out of 41) of the neonates with either full or partial defect in the G6PD enzyme were admitted with significant NJ compared with 27.5% (44 out of 160) without G6PD enzyme defect with a statistically significant difference in the proportions (p = 0.009). Regarding the proportion of the 201 patients who had G6PD tested, 20% (41) had either full or partial defect of the enzyme.

Further, we determined the neonatal predictors of NJ severity, and the results are presented in Table 3. Neonates admitted more than a day after birth were 3.09 times more likely to present with significant NJ compared to those who were admitted within the first day of birth (OR=3.09, 95% CI: 1.76–5.44), 2.45 when adjusted for by the other parameters (aOR=2.45, 95% CI: 1.26–4.77). Additionally, neonates who had either full or partial defect of the G6PD enzyme were 2.51 times more likely to present with significant NJ compared to neonates who had no defect in the enzyme (OR=2.51, 95% CI: 1.24–5.08) and 2.21 when adjusted (aOR=2.21, 95% CI: 1.03–4.71).

**Table 3:** Determinants of NJ severity at admission at the Paediatric Ward and NICU at Cape Coast Teaching Hospital during the study period.

		cOR (95% CI)	*p*-value	aOR (95% CI)	*p*-value
Variable	Category				
Age at admission	≤ 24 hours	1 (ref)	< 0.001	1 (ref)	0.009
	> 24 hours	3.09 (1.76, 5.44)		2.45 (1.26, 4.77)	
Sex	Female	1 (ref)	0.45	1 (ref)	0.94
	Male	1.24 (0.71, 2.18)		0.98 (0.50, 1.90)	
Birthweight	Normal	1 (ref)		1 (ref)	
	Low	0.58 (0.33, 1.05)	0.073	1.22 (0.47, 3.19)	0.69
	Macrosomia	1.29 (0.35, 4.80)	0.70	2.42 (0.52, 11. 21)	0.26
Gestational Age	Term	1 (ref)	0.003	1 (ref)	0.11
	Preterm	0.41 (0.23, 0.74)		0.45 (0.17, 1.20)	
G6PD status	No defect	1 (ref)	0.010	1 (ref)	0.041
	Full/Partial defect	2.51 (1.24, 5.08)		2.21 (1.03, 4.71)	

## Discussion

NJ is associated with significant morbidity and mortality in neonates, and it is an important indication for the readmission of neonates globally. Thus, it is imperative that medical practitioners, especially in LMICs where NJ and complications are prevalent, be cognisant of risk factors in neonates that can predispose them to developing severe NJ. Although severe NJ is generally defined as TSB concentration greater than 342–428 micromol/L, we, however, employed the threshold of > 213 micromol/L define NJ severity because this threshold has mainly been employed for the treatment in African countries where prompt management to forestall the development of NJ complications has become imperative [2]. Although there are no epidemiological studies based on this definition in literature, Amadi *et. al*. employed the definition to stratify neonates to compare overhead and total body phototherapy in Nigeria [3]. Additionally, severe NJ was defined at a threshold lower than the 213micromol/L threshold, that is, 205 micromol/L to assess the risk of neonates to naphthalene exposure [33].

In our study, the following were found to be significant determinants of NJ severity: age at admission and G6PD status. Neonates developing NJ within a day of life is less common compared with after a day of life, however, this phenomenon tends to be associated with significant risk factors in addition to being a major risk factor for the development of severe NJ and BIND subsequently [34,35]. Approximately 57% of the neonates were admitted within the first day of birth, however, because the neonates in our study were not followed up to determine whether they developed long-term sequelae, we were unable to ascertain whether early onset of NJ was associated with long-term effects. In our study, neonates admitted to the NICU after 24 hours of life were, however, over 2 times likely to have significant NJ compared with those admitted within the first 24 hours of life. In a country where only 23% and 30% of newborns receive examination within 48 hours and 1 week respectively after birth, the likelihood of most newborns with NJ being missed is high especially in dark-skinned individuals [36]. Exclusive breastfeeding is associated with an increased risk of developing NJ in neonates and the unexpected result of preterm neonates having a lower risk for NJ may stem from breastfeeding practices in Ghanaian NICUs. Healthcare staff tend to base enteral feeding on several factors, including gestational age, with avoidance of enteral feeding in preterm neonates because of poor suckling reflexes [37]. Thus, the term neonates, who are likely to be exclusively breastfed may be at a higher risk of developing breastfeeding NJ compared with preterm neonates who tend to be wholly or partially fed parenterally within the few hours of birth. Nonetheless, the phenomenon would need to be investigated, as studies elsewhere have not reported similar findings [5]. Irrespective of the lower prevalence of significant NJ in preterm neonates in our study, they are still at a greater risk of bilirubin encephalopathy as the underdeveloped brain is more susceptible to unconjugated bilirubin neurotoxicity and transport across the blood-brain barrier [13]. Additionally, conditions more prevalent in preterm neonates, including hypoalbuminaemia (increased unbound unconjugated bilirubin), hypoxia, sepsis and acidosis also contribute to increased susceptibility [13].

G6PD enzyme defect with a global prevalence of 4.6%, more prevalent in sub-Saharan Africa and Asia, is an important risk factor for the development of NJ [5,35,38,39]. In Ghana, G6PD prevalence is reported to range from 10% to 23% and is a determinant of NJ development in neonates [24,25,40,41]. The G6PD enzyme produces NADPH an important cofactor in the reduction of cellular peroxide by the glutathione and thioredoxin reductases, preventing the peroxidation of red blood cell plasma membrane from intracellular peroxide accumulation [42–44]. In our study, 20% of the neonates had G6PD defect (full or partial), a prevalence much higher than the 5.1% and 11% reported by other studies undertaken in southern Ghana [25,27]. Additionally, the prevalence was higher compared with pooled data of studies undertaken in Iran and the 2.3% prevalence reported from India [9,45]. In sub-Saharan Africa, the prevalence in our study was in the range of 11.8% to 30.7% prevalence of G6PD defect in NJ reported from in Nigeria [46,47]. Certain practices in Ghana and some West African countries including the usage of naphthalene mothballs as drinking water purifier and herbal medications which can cause haemolysis especially in G6PD enzyme deficient individuals could put neonates at risk of developing NJ [33,48,49]. In fact, there is a previous report of detection of naphthalene in neonatal and maternal blood of neonates presenting with NJ [33].

NJ is an important indicator for admissions to the paediatric and NICU wards in Ghana. There are international guidelines for assessing newborns at risk of NJ development and how to manage them [50–52]. With the risk factors for NJ common among neonates in Ghana, local guidelines for managing NJ ought to be developed to manage these neonates. To achieve this aim, studies focusing on risk factors for the development of severe NJ, effective management strategies and the evolution of neonatal NJ to KSD sequelae need to be undertaken across the country.

### Limitations

The study was a retrospective review of patient records, therefore, some data needed for analyses on the risk factors associated with severe NJ were not available including neonatal co-morbid such as sepsis, maternal characteristics such as blood groupings and peripartum characteristics such delivery complications were not available. Data on other risks factors including breast feeding practices and potential household risk-associated practices were also not available. Additionally, the burden of acute and chronic complications associated with NJ at the clinic could not be determined because of unavailability of data in the electronic database. The results from this study cannot be generalised to the general population as the study was undertaken in a teaching hospital which serves as a referral health facility.

## Conclusions

The study revealed that G6PD deficiency and age at admission or presentation are significant risk factors for the development of significant NJ. It is, therefore, imperative for both families and healthcare providers to understand the various risk factors associated with NJ development and place emphasis on prevention of modifiable causes, early detection and proper management to reduce the morbidity and mortality challenges that it presents with. The study strengthens the evidence for the mandatory assessment of neonates for G6PD. Neonatal screening for G6PD in addition to maternal and neonatal blood grouping (ABO, Rhesus) tests and inclusion of signs and symptoms and preventive measures of NJ in antenatal booklets will be important for the reduction in the incidence of severe NJ at health centres in Ghana.

## Supporting information

S1 File Anonymized neonatal jaundice data. (XLSX)
